# Supporting Self-Direction in Social and Daily Life Contexts Among Vulnerable Older Adults: A Protocol for an Integrative Review and Concept Analysis

**DOI:** 10.3390/bs15121718

**Published:** 2025-12-11

**Authors:** Golnaz Atefi, Lieve Roets-Merken, Maud J. L. Graff

**Affiliations:** 1 Department of IQ Health, Radboud University Medical Centre, 6525 GA Nijmegen, The Netherlands; 2 Radboud Alzheimer Centre, 6525 GA Nijmegen, The Netherlands; 3 Department of Rehabilitation, Radboud University Medical Centre, 6525 GA Nijmegen, The Netherlands

**Keywords:** self-direction, older adults, dementia, vulnerable, meaningful activities, social participation

## Abstract

Objectives: This study aims to provide conceptual clarity on self-direction support in the care of vulnerable older adults, particularly those with dementia. It focuses on how self-direction is supported in meaningful daily activities and social participation. The goal is to define and operationalize the concept by identifying its key attributes, antecedents, and consequences across care contexts. Methods: A two-phase approach will be used. First, an integrative review will synthesize empirical evidence from gerontology, occupational therapy, psychology, nursing, and health ethics to examine current conceptualizations and practices. Second, a concept analysis will explore the theoretical structure of self-direction support. Findings will be synthesized into a conceptual framework. Expected outcomes: This study is expected to provide a clearer conceptual framework outlining the core components of self-direction as described in existing literature. This framework will define key attributes, identify influencing factors, and propose measurable indicators. The framework aims to guide professionals in balancing autonomy, safety, and care needs. Conclusions: As this is a study protocol, no results are presented; findings will be reported in the forthcoming review. The anticipated outcomes are expected to contribute to theory and practice by framing self-direction within social health. The framework may inform future research, policy, and intervention development to strengthen self-direction in meaningful activities and participation among vulnerable older adults. Further validation across settings and cultural contexts will be required.

## 1. Introduction

### 1.1. Rationale

As populations age globally, a growing number of older adults, especially those living with chronic conditions such as dementia, face increasing vulnerabilities that extend beyond physical health (Livingston et al., 2020). Dementia, a progressive neurodegenerative disorder marked by continuous cognitive decline, gradually limits individuals’ ability to live independently (Livingston et al., 2020). With the aging population and the rising global prevalence of dementia, the associated healthcare costs for vulnerable older adults are projected to pose a major societal challenge (Gauthier et al., 2022). However, this burden is not solely clinical or economic. A substantial portion of care for vulnerable older adults with and without dementia occurs within communities, predominantly provided by family members (Schulz et al., 2020). This shifting landscape raises not only medical and logistical concerns but also deeper questions about how these vulnerable older adults can continue to maintain or retain autonomy and self-direction in daily life.

Particularly in the context of dementia and other chronic or long-term conditions, the ability to sustain self-direction becomes increasingly constrained as functional decline progresses. With growing vulnerability and dependence, the boundaries of how and to what extent individuals are able and enabled to make choices about their daily activities, values, preferences, and goals may become blurred. This ambiguity can create challenges for both formal and informal caregivers, whether in home-based or institutional settings. Nevertheless, self-direction is a critical determinant of quality of life and life satisfaction. It requires continuous attention to ensure that care provision remains balanced, aligning the perspectives of caregivers and vulnerable older adults in identifying unmet needs and collaboratively determining appropriate ways to address them.

A systematic review of available interventions for supporting self-direction in people with dementia shows that such interventions remain considerably limited and are often focused on advance care planning rather than on active day-to-day living and social participation in meaningful activities (Döpp et al., 2021). Further research is needed to clarify how self-direction support for vulnerable older adults, with or without dementia, is conceptualized across professional domains, how it is experienced by older adults themselves, and how it can be effectively implemented in practice (Döpp et al., 2021). In response, a global initiative has focused on innovative research to address and enhance eldercare throughout the entire aging and dementia trajectory (Monnet et al., 2023). In this study, we use a working description of self-direction as the way in which vulnerable older adults are able to express preferences, make choices, and maintain influence over aspects of their everyday and social lives. This is not intended as a definitive definition; the concept will be clarified and refined through the planned integrative review and concept analysis. By outlining this provisional description, we indicate the general scope of the construct we seek to examine.

### 1.2. Self-Direction in Everyday and Social Life

With aging, the likelihood of experiencing social isolation increases, often due to a combination of physical and cognitive limitations and the gradual reduction in one’s social network (Steverink & Lindenberg, 2006; Brearley, 2023; Sachdev, 2021). For older adults, fulfilling social needs is critical to their overall well-being. These needs include social connections, engagement in meaningful activities, and managing daily life (Ten Bruggencate et al., 2018, 2019). In the context of dementia, these social needs become more pronounced. As the condition progresses, people with dementia and their families may face greater demands and fewer available social resources or opportunities for connection (Sachdev, 2021; Liao et al., 2024). These factors contribute to increased vulnerability to social isolation and reduced engagement in meaningful daily activities among vulnerable older adults (Van der Roest et al., 2009). On the other hand, in the absence of clear boundaries regarding the appropriate extent and nature of support, formal and informal caregivers may (un)intentionally take control over certain aspects of social functioning, which can lead to a mismatch between the support provided and the vulnerable older adult’s actual capacities or willingness to engage, potentially undermining their autonomy and self-direction. As a result, many older adults, particularly those living with dementia, report persistent gaps in balanced support. A common unmet need in this population is the lack of opportunities for meaningful daily engagement and the maintenance of social connections (Van der Roest et al., 2009). Evidence increasingly supports that it is both possible and beneficial to address these needs in dementia and eldercare settings. Tailored approaches that support engagement in meaningful activities, social participation, and connection can lead to improvements in psychosocial and behavioural outcomes, ultimately promoting better functioning and enhanced well-being (Oyebode & Parveen, 2019; Graff et al., 2006, 2007). This drive for balanced support is also essential for person-centered care and reflects a broader understanding of well-being in later life. Existing literature shows that terms related to autonomy, self-management, empowerment, participation, and shared decision-making are sometimes used interchangeably or with overlapping meanings. The extent to which these concepts relate to, differ from, or fall within the scope of self-direction remains unclear, and this will be explored further in the planned review.

### 1.3. Social Health as the Conceptual Frame

Although the idea of supporting self-direction appears across gerontology, occupational therapy, nursing, and social care, the term itself is used inconsistently and often without clear conceptual boundaries. This lack of clarity limits how self-direction support can be recognised or applied in practice. The planned integrative review and concept analysis will address this gap by examining how the concept is currently described and used.

Recent research in dementia and elder care increasingly uses the concept of social health as a framework to understand how older adults can maintain autonomy and engagement in the face of physical or cognitive decline (Ten Bruggencate et al., 2018, 2019). Social health refers to the ability to participate in daily and social activities, maintain relationships, and manage everyday life through adaptive strategies. It also calls for environments that support autonomy and dignity, even amid age-related challenges such as dementia (Dröes et al., 2017; Vernooij-Dassen et al., 2018). Social health emphasizes strengths rather than limitations, highlighting the need to balance vulnerability with support that enables independence and engagement. This concept emphasizes the importance of balancing age-related vulnerabilities, such as dementia, with the need to support and strengthen the remaining abilities and capacities of older adults (Dröes et al., 2017; Vernooij-Dassen et al., 2018). Yet, how to support self-direction effectively, within the broader framework of social health, remains conceptually unclear.

Balanced support, where autonomy is maintained alongside safety and guidance, may promote agency, self-regulation, and overall functioning (Oyebode & Parveen, 2019; Graff et al., 2006, 2007). When this support comes from caregivers and professionals, it may encourage self-direction, autonomy, and a sense of agency necessary for optimal social health and functioning of vulnerable older adults. However, evidence on how such self-direction support is defined and practiced remains fragmented across disciplines such as occupational therapy, nursing, gerontology, psychology, and health ethics. This underscores the need for a clear, evidence-based framework that defines the necessary support for self-direction and provides practical tools for its implementation in daily and social activities. This study addresses this gap by developing a clear, evidence-based conceptual framework that defines self-direction support and offers guidance for its application in daily and social care contexts, using social health as the guiding theoretical lens.

### 1.4. Present Study

Supporting self-direction is increasingly recognised as important, but clear conceptual boundaries are still missing. In a social context, supporting self-direction is increasingly seen as a core, yet underdefined element of eldercare. Given the overlaps described above and the absence of a unified description, there is a need for conceptual work that will clarify how self-direction is understood and supported in everyday and social life. The present study aims to address this need by systematically examining existing literature and identifying the key attributes, antecedents, and consequences of the concept.

This study aims to clarify self-direction support in the care of vulnerable older adults with a particular focus on dementia. Any references to application or practice refer to illustrating how the concept appears in everyday contexts. A two-phase approach will address the research question: How is self-direction support in daily activities understood and implemented for older adults with and without dementia? First, an integrative review will synthesize existing knowledge to identify how self-direction support is conceptualized and applied across disciplines. Second, a concept analysis will identify the key attributes, antecedents, and consequences of self-direction support, creating a clear operational definition and conceptual framework. This approach ensures the final framework is both empirically grounded and theoretically robust, offering practical guidance for caregivers and professionals in supporting autonomy while balancing safety in daily and social activities.

## 2. Materials and Methods

The review protocol will also be registered with PROSPERO, the international database for systematic review protocols. This study employs a structured methodological approach that combines an integrative review with concept analysis to provide a comprehensive understanding of self-direction support in meaningful activities, framed within the broader context of social health. This combination allows for both empirical synthesis and theoretical clarification, ensuring conceptual robustness and practical relevance.

### 2.1. Integrative Review

The first phase of the study will consist of an integrative review conducted in accordance with the methodology proposed by Whittemore and Knafl (2005). Furthermore, the PRISMA-S guidelines will be followed for the integrative review phase to ensure comprehensive and transparent reporting of the systematic search and synthesis of existing literature. In line with Whittemore and Knafl’s methodology for integrative reviews, this study will follow five systematic stages to enhance methodological rigour and transparency. Table 1 summarizes these stages and outlines how they will be applied in the present review.

The review will synthesize existing knowledge across disciplines to explore patterns, inconsistencies, and practical implications of self-direction support in elderly care. A systematic search of relevant literature will be conducted across multiple databases, including PubMed, CINAHL, PsycINFO, and Google Scholar. The search terms will be designed to capture terminological variations in self-direction and related social health concepts, and will be adapted to the syntax and controlled vocabulary of each database (see Supplementary Material S1). Given the limited number of empirical publications identified in earlier work, including those focusing mainly on advance care planning (Döpp et al., 2021), we anticipate that research directly addressing self-direction in daily and social life may be sparse. For this reason, the review will also examine policy documents, professional guidelines, and grey literature to determine how the concept is currently described and applied across sectors.

Inclusion criteria will cover publications including quantitative and qualitative studies, theoretical papers, and systematic reviews addressing support for self-direction among older adults, particularly in relation to daily functioning and social engagement. Relevant sources such as reports and guidelines will be included to minimize publication bias, ensuring a thorough understanding of the topic.

Specifically, publications will be eligible for inclusion if they: (i) are published in English; (ii) focus on vulnerable older adults (typically aged 65 or above) with any chronic or long-term conditions including those receiving long-term care or home-based care; (iii) discuss the concept of self-direction or closely related constructs in the context of daily living activities and social participation; (iv) involve interventions, care practices, or support services.

Studies not focused on aging populations or limited to medical decision-making will be excluded. Publications will be excluded if they: (i) are focusing on advance care planning; (ii) focus solely on acute medical decision-making without addressing broader aspects of daily or social life; (iii) provide insufficient conceptual or practical insight into self-direction or its support mechanisms. Titles and abstracts will be screened independently by two researchers, followed by full-text screening. Discrepancies will be resolved through consensus. Data will be extracted and categorized by theme, methodology, and outcomes. Regarding self-direction support, data extraction will focus on identifying how self-direction is described, supported, or operationalised within each publication. Extracted information will be organised in structured matrices to allow systematic comparison across diverse settings. This approach will allow to map similarities, differences, and gaps in how self-direction is addressed across fields. Findings from the integrative review will inform the concept analysis, enabling cross-validation between theory and practice.

A quality appraisal process will be conducted in the review to clarify the methodological strengths and limitations of the included materials. Appraisal tools will be selected based on the type of evidence reviewed. Given the expected diversity of sources including empirical studies, theoretical papers, and grey literature, multiple tools may be used, such as Authority, Accuracy, Coverage, Objectivity, Date, Significance (AACODS) checklist (Tyndall, 2010), Critical Appraisal Skills Programme (CASP) (Long et al., 2020), or tools from Joanna Briggs Institute (Munn et al., 2019), depending on the nature of the literature.

### 2.2. Concept Analysis

The second phase of the study follows the concept analysis method developed by Walker and Avant (2005), which is designed to systematically clarify and define key theoretical constructs. Building on the findings of the integrative review, the concept analysis will focus on the theoretical underpinnings of self-direction support as it relates to social participation and daily life among vulnerable older adults. The analysis begins with the selection of the concept and establishing its purpose: to develop a theoretically sound and practically applicable definition of self-direction support. An extensive exploration of the various uses of the concept will be undertaken, drawing from dictionaries, healthcare policies, professional guidelines, and academic literature spanning disciplines such as gerontology, occupational therapy, psychology, and medical ethics. Closely related constructs (e.g., autonomy, empowerment, participation, self-management, decision-making) will be identified through terminology mapping during screening and extraction. For each construct, we will document how it is described and whether it is positioned as overlapping with, distinct from, or substituting the term self-direction. This will support a clearer understanding of conceptual boundaries, which will be further examined in the concept analysis and identification of key attributes that consistently characterize self-direction support. In accordance with Walker and Avant’s framework, this process will explicitly follow the eight steps: (1) selecting the concept; (2) determining the purpose of the analysis; (3) identifying all uses of the concept; (4) defining the attributes; (5) constructing model cases that include all attributes; (6) developing additional cases such as borderline, related, and contrary cases to delineate boundaries; (7) identifying antecedents and consequences; and (8) defining empirical referents as measurable indicators. In the context of the Walker and Avant method, identifying antecedents and consequences is to clarify the conceptual boundaries of the term. Antecedents refer to the conditions or situations that need to be present for self-direction to occur, whereas consequences illustrate what typically follows when self-direction is supported or absent.

Once the defining attributes are established, model cases, contrary cases, and borderline cases will be developed to illustrate the concept’s various applications and limitations. These illustrative cases will draw on descriptions from the literature to show how the concept is understood and applied across contexts. Subsequently, antecedents and consequences will be identified. Antecedents include capacity, access to supportive environments, and the availability of resources that facilitate decision-making. The consequences of effective self-direction support as part of social health will be examined in terms of improved quality of life and relationships, enhanced social participation, and reduced caregiver burden.

Expert consultation will be conducted through a structured focus group. A purposive sample of experts in gerontology, occupational therapy, dementia care, and social health will be invited to review the preliminary attributes, antecedents, and consequences identified in the review. The focus group will include 6–10 participants and will follow a semi-structured format to allow systematic discussion of the emerging conceptual structure. Participants will be asked to comment on the clarity, completeness, disciplinary relevance, and practical applicability of the developing concept. The session will be audio-recorded, summarised, and analysed to refine the conceptual boundaries and strengthen the final framework. In this phase, it will be essential to ensure transparency and consistency, particularly regarding how sources are selected, how consensus is reached on key attributes, and whether expert feedback can be incorporated to support the interpretation of the results. Finally, empirical referents will be identified to allow for future measurement and implementation, such as verbal and non-verbal expressions of autonomy, active participation in planning activities, and the use of adaptive strategies to maintain control in daily life. The integration of findings from both methodological phases will result in a unified conceptual framework that defines self-direction support and outlines how it can be recognized and applied in practice. This framework will serve as a foundation for future research, policy development, and practical interventions aimed at enhancing autonomy and functioning among vulnerable older adults with and without dementia.

## 3. Discussion

As this is a protocol paper, no empirical findings are available to present or interpret. Thus, this discussion is based on existing literature and identified gaps, and therefore reflects the anticipated contributions of the planned study rather than empirical findings. This study aims to bridge both conceptual and practical gaps in understanding how self-direction is supported in the care of vulnerable older adults, particularly those living with dementia. By integrating insights from empirical research and theoretical exploration, it aims to clarify the meaning, scope, and application of self-direction support within the broader framework of social health. In doing so, it addresses the complexity of promoting more self-direction, autonomous participation, and meaningful engagement in later life. The preliminary synthesis will aim to demonstrate that while support for self-direction is increasingly recognized as important, the nature and extent of such support remain inconsistently defined and applied across disciplines and care settings Findings from the integrative review and concept analysis will inform the development of a conceptual framework that captures the core attributes, antecedents, and consequences of self-direction support, as well as the contextual factors influencing its implementation. This framework is intended to guide future interventions and policies by emphasizing the importance of balanced support, support that upholds autonomy and self-direction while also addressing safety and care needs in daily and social life. A thorough discussion of actual results and their implications will be provided once the integrative review and concept analysis are completed. Further research will be needed to validate this framework and explore its applicability across diverse care settings and cultural contexts.

## Figures and Tables

**Table 1:** Overview of the integrative review methodology (adapted from Whittemore & Knafl, 2005).

Stage	Description (Whittemore & Knafl)	Application in This Review
1- Problem identification	Clearly define the problem, concepts, target population, and variables of interest to provide focus and boundaries for the review.	The review will address how self-direction support in daily activities and social participation is understood and implemented for vulnerable older adults, particularly those with dementia.
2- Literature search	Conduct a comprehensive and systematic search across multiple sources to minimize bias.	Searches will be conducted in scientific databases, complemented by ancestry searching, hand-searching, and grey literature sources
3- Data evaluation	Assess the quality and relevance of diverse empirical and theoretical sources using fit-for-purpose appraisal tools.	Quality of included publications will be appraised using valid tools. Quality appraisal will be recorded and used to weight contributions during analysis rather than for strict exclusion.
4- Data analysis	Extract, reduce, display, and compare data iteratively to identify patterns, themes, relationships, and higher-level abstractions.	Data will be coded into matrices to capture definitions, attributes, antecedents, consequences, and outcomes of self-direction support.
5- Presentation	Provide a comprehensive and transparent synthesis that links conclusions to primary sources and outlines implications.	Results will be presented in narrative and diagrammatic forms to capture the breadth and depth of the concept. Limitations of the review will be explicitly reported.

## Data Availability

No new data were created or analyzed in this study.

## Supplementary Materials

The following supporting information can be downloaded at: https://www.mdpi.com/article/10.3390/bs15121718/s1. File S1: Search strategy.

## References

[B1-behavsci-15-01718] Brearley C. P. (2023). Social work, ageing and society.

[B2-behavsci-15-01718] Döpp C. M., Drenth H., Verkade P. J., Francke A. F., Van Der Heide I. (2021). Interventions for improving self-direction in people with dementia: A systematic review. BMC Geriatrics.

[B3-behavsci-15-01718] Dröes R. M., Chattat R., Diaz A., Gove D., Graff M., Murphy K., Verbeek H., Vernooij-Dassen M. J. F. J., Clare L., Johannessen A., Roes M. (2017). Social health and dementia: A European consensus on the operationalization of the concept and directions for research and practice. Aging & Mental Health.

[B4-behavsci-15-01718] Gauthier S., Webster C., Servaes S., Morais J. A., Rosa-Neto P. (2022). World Alzheimer report 2022: Life after diagnosis: Navigating treatment, care and support.

[B5-behavsci-15-01718] Graff M. J., Vernooij-Dassen M. J., Thijssen M., Dekker J., Hoefnagels W. H., OldeRikkert M. G. (2007). Effects of community occupational therapy on quality of life, mood, and health status in dementia patients and their caregivers: A randomized controlled trial. The Journals of Gerontology Series A: Biological Sciences and Medical Sciences.

[B6-behavsci-15-01718] Graff M. J., Vernooij-Dassen M. J., Thijssen M., Dekker J., Hoefnagels W. H., Rikkert M. G. O. (2006). Community based occupational therapy for patients with dementia and their care givers: Randomised controlled trial. BMJ.

[B7-behavsci-15-01718] Liao X., Wang Z., Zeng Q., Zeng Y. (2024). Loneliness and social isolation among informal carers of individuals with dementia: A systematic review and meta-analysis. International Journal of Geriatric Psychiatry.

[B8-behavsci-15-01718] Livingston G., Huntley J., Sommerlad A., Ames D., Ballard C., Banerjee S., Brayne C., Burns A., Cohen-Mansfield J., Cooper C., Costafreda S. G. (2020). Dementia prevention, intervention, and care: 2020 report of the Lancet Commission. The Lancet.

[B9-behavsci-15-01718] Long H. A., French D. P., Brooks J. M. (2020). Optimising the value of the critical appraisal skills programme (CASP) tool for quality appraisal in qualitative evidence synthesis. Research Methods in Medicine & Health Sciences.

[B10-behavsci-15-01718] Monnet F., Dupont C., Pivodic L. (2023). In global approaches to dementia research, do not forget care. Nature Medicine.

[B11-behavsci-15-01718] Munn Z., Aromataris E., Tufanaru C., Stern C., Porritt K., Farrow J., Lockwood C., Stephenson M., Moola S., Lizarondo L., McArthur A. (2019). The development of software to support multiple systematic review types: The Joanna Briggs Institute System for the Unified Management, Assessment and Review of Information (JBI SUMARI). JBI Evidence Implementation.

[B12-behavsci-15-01718] Oyebode J. R., Parveen S. (2019). Psychosocial interventions for people with dementia: An overview and commentary on recent developments. Dementia.

[B13-behavsci-15-01718] Sachdev P. S. (2021). Restriction of activities, social isolation, and dementia. International Psychogeriatrics.

[B14-behavsci-15-01718] Schulz R., Beach S. R., Czaja S. J., Martire L. M., Monin J. K. (2020). Family caregiving for older adults. Annual Review of Psychology.

[B15-behavsci-15-01718] Steverink N., Lindenberg S. (2006). Which social needs are important for subjective well-being? What happens to them with aging?. Psychology and Aging.

[B16-behavsci-15-01718] Ten Bruggencate T., Luijkx K. G., Sturm J. (2018). Social needs of older people: A systematic literature review. Ageing & Society.

[B17-behavsci-15-01718] Ten Bruggencate T., Luijkx K. G., Sturm J. (2019). When your world gets smaller: How older people try to meet their social needs, including the role of social technology. Ageing & Society.

[B18-behavsci-15-01718] Tyndall J. (2010). AACODS checklist.

[B19-behavsci-15-01718] Van der Roest H. G., Meiland F. J., Comijs H. C., Derksen E., Jansen A. P., Van Hout H. P., Jonker C., Dröes R. M. (2009). What do community-dwelling people with dementia need? A survey of those who are known to care and welfare services. International Psychogeriatrics.

[B20-behavsci-15-01718] Vernooij-Dassen M., Moniz-Cook E., Jeon Y. H. (2018). Social health in dementia care: Harnessing an applied research agenda. International Psychogeriatrics.

[B21-behavsci-15-01718] Walker L. O., Avant K. C. (2005). Strategies for theory construction in nursing.

[B22-behavsci-15-01718] Whittemore R., Knafl K. (2005). The integrative review: Updated methodology. Journal of Advanced Nursing.

