# Supplementary material for: Supporting Self-Direction in Social and Daily Life Contexts Among Vulnerable Older Adults: A Protocol for an Integrative Review and Concept Analysis
Source: Behav Sci (Basel). 2025 Dec 11;15(12):1718. doi: 10.3390/bs15121718 (PMC12729509; doi:10.3390/bs15121718)
Supplement: Supplementary file 1 [file behavsci-15-01718-s001.zip › behavsci-3910139-supplementary.pdf]

Supplementary Material S1.

An overview of the search strategy.

("Dementia"[MeSH Terms] OR "alzheimer*"[Title/Abstract] OR "dementi*"[Title/Abstract] OR "cognitive impair*"[Title/Abstract] OR "cognitive decline*"[Title/Abstract] OR "neurodegenerative disorders*"[Title/Abstract] OR "vulnerab*"[Title/Abstract] OR "frail*"[Title/Abstract] OR "multimorbid*"[Title/Abstract] OR "chronic illness"[Title/Abstract]) AND ("older adults"[Title/Abstract] OR "Elderly"[Title/Abstract] OR "aging population*"[Title/Abstract] OR "vulnerable older adults*"[Title/Abstract] OR "olde*"[Title/Abstract]) AND ("Self-Management"[MeSH Terms] OR "Decision Making"[MeSH Terms] OR "Personal Autonomy"[MeSH Terms] OR "self manage*"[Title/Abstract] OR "selfmanag*"[Title/Abstract] OR "self direct*"[Title/Abstract] OR "selfdirect*"[Title/Abstract] OR "decision making*"[Title/Abstract] OR "decisionmaking*"[Title/Abstract] OR "sdm"[Title/Abstract] OR "autonomy"[Title/Abstract] OR "mastery"[Title/Abstract] OR "empower*"[Title/Abstract] OR "patient participation"[Title/Abstract] OR "patient engagement"[Title/Abstract] OR "patient involvement"[Title/Abstract]) AND ("social health*"[Title/Abstract] OR "social participation*"[Title/Abstract] OR "meaningful activities*"[Title/Abstract] OR "social functioning*"[Title/Abstract] OR "social engagement*"[Title/Abstract] OR "social interaction*"[Title/Abstract] OR "social inclusion*"[Title/Abstract] OR "daily life"[Title/Abstract] OR "everyday life"[Title/Abstract] OR "daily functioning"[Title/Abstract] OR "social roles"[Title/Abstract])
